# Deciphering Uncoupling Proteins in Cellular Homeostasis and Metabolic Health

**DOI:** 10.7150/ijbs.129896

**Published:** 2026-03-17

**Authors:** Yu-Ting Liu, Ya-Jia Ding, Hong-Liang Qiu, Yan Che, Qi-Zhu Tang

**Affiliations:** 1 Department of Cardiology, Renmin Hospital of Wuhan University, Wuhan 430060, RP China.; 2 Hubei Key Laboratory of Metabolic and Chronic Diseases, Wuhan 430060, RP China.

**Keywords:** Uncoupling proteins, Thermoregulation, Cellular homeostasis, Metabolic health, Cardiovascular disease, Tumor

## Abstract

Uncoupling proteins (UCPs) are transmembrane proteins located in the inner membrane of mitochondria. They can reduce the efficiency of ATP synthesis and promote heat production by mediating the uncoupling oxidative phosphorylation process. Different subtypes of UCPs have distinct tissue distributions and functional characteristics, involving various biological processes including temperature regulation, energy balance, signal transduction, oxidative stress, and the inflammatory response. In recent years, many studies have shown the potential value of UCPs in the prevention and treatment of metabolic diseases such as obesity, diabetes, cardiovascular diseases, neurological diseases, and tumors. Importantly, multiple evidence reveals that innovative therapies targeting uncoupling proteins will show broad application prospects in the future. This review of recent research on UCPs aims to provide a direction for exploring the molecular mechanisms of cellular homeostasis and intervention strategies for metabolic diseases.

## Introduction

**Figure 1:**
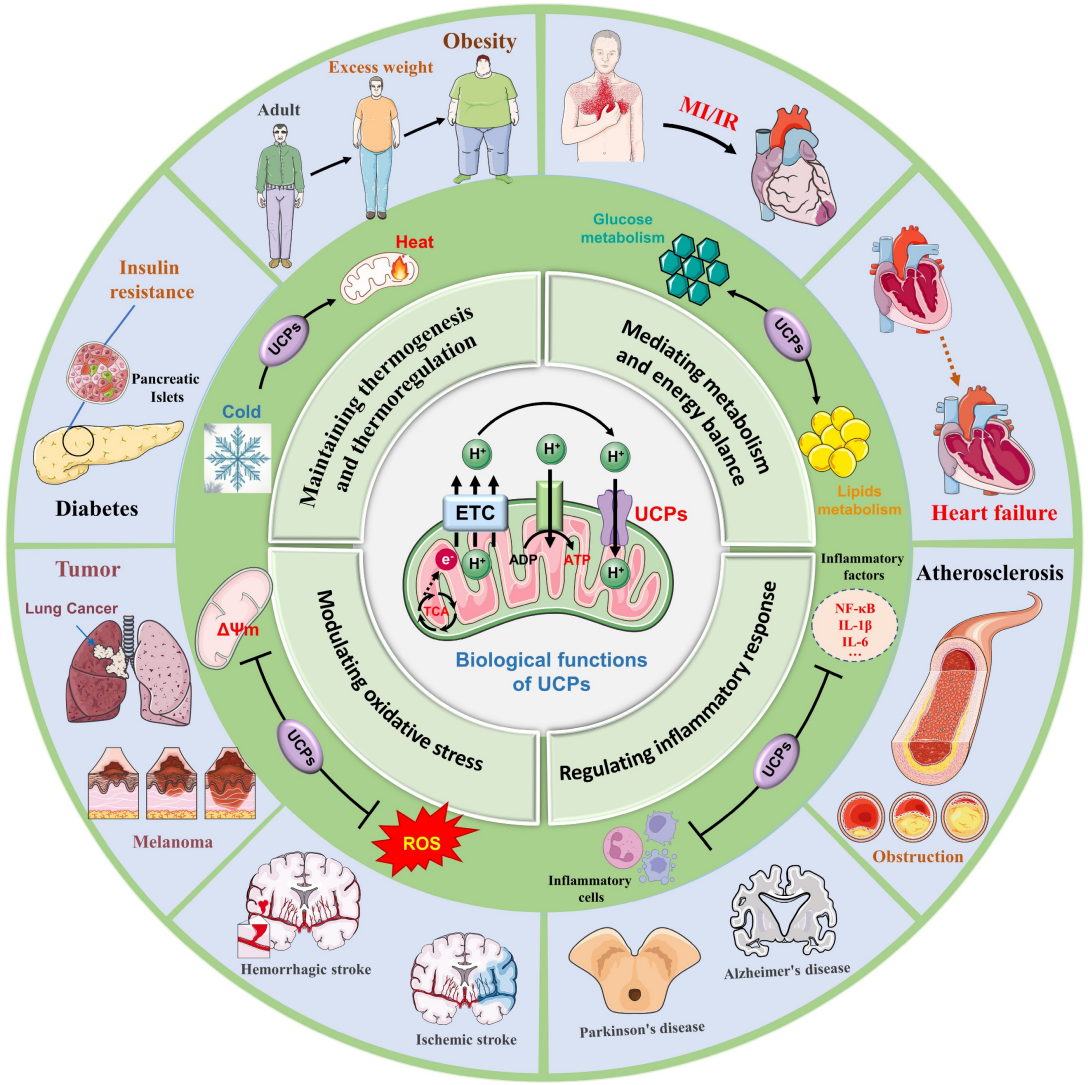
** Biological processes and disease crosstalk in UCPs.** UCPs are a family of transmembrane proteins located on the inner membrane of mitochondria. They are widely present in almost all cell types and play a key role in the proton leak channel transmitted by the mitochondrial TCA cycle. UCPs are widely involved in various biological processes, including (1) Maintaining thermogenesis and thermoregulation, (2) Mediating metabolism and energy balance, (3) Modulating oxidative stress, and (4) Regulating inflammatory response. Furthermore, UCPs serve a significant part in obesity and diabetes, cardiovascular disease, neurological illnesses, and tumor growth, making them an important target for disease preventive and therapy efforts. TCA, tricarboxylic acid cycle; ADP, adenosine diphosphate; ROS, reactive oxygen species; MI, myocardial infarction; IR, ischemia-reperfusion, NF-kB, nuclear factor-κB; IL-1β, nterleukin-1 beta; IL-6, nterleukin-6.

Uncoupling proteins (UCPs) are a class of transmembrane proteins located in the inner mitochondrial membrane and belong to the mitochondrial carrier protein family 1. Previous studies have shown that electron transfer in the respiratory chain drives protons to be pumped out of the mitochondrial matrix into the membrane space, thereby forming a proton concentration gradient. Protons flow back to the matrix through ATP synthase to drive the generation of ATP in the mitochondrial oxidative phosphorylation process 2, 3. UCPs form proton leak channels, which prevent the proton motive force (PMF) in the oxidative phosphorylation process from longer being completely used solely for ATP synthesis, but instead allowing some of the PMF to be released in the form of heat 4. Multiple subtypes of UCPs have different localization distributions and structural functions, and are found mainly in brown adipose tissue (BAT), white adipose tissue (WAT), skeletal muscle, myocardium and the brain 5. UCP1 is the main uncoupling protein, and found mainly in BAT, it is responsible for nonshivering thermogenesis, and is highly important for regulating energy metabolism and maintaining body temperature 6. UCP2 and UCP3 are related to obesity, fatty acid metabolism and blood sugar regulation, while UCP4 and UCP5 are distributed mainly in brain nerve tissue and are closely related to the pathogenesis of neurodegenerative diseases 7** (Fig. 1)**.

Given that UCPs are involved in mediating thermoregulation, energy balance, signal transduction, oxidative stress, and inflammatory responses, UCPs are key regulators of cellular energy metabolism and redox homeostasis, and also are core molecular switches for maintaining overall cellular homeostasis. Numerous studies have explored the interactions of UCPs with other metabolic pathways, which is conducive to clarifying the mechanism of action of uncoupling proteins in obesity, cardiovascular disease, neurodegenerative diseases, and tumors 8-10. Recent studies have revealed that UCP1 prevents acute kidney injury (AKI) by inhibiting oxidative stress, and that endothelial UCP2 is a mechanosensitive inhibitor of atherosclerosis 11, 12. In addition, UCP3 is closely related to myocardial susceptibility to ischaemia/reperfusion injury, and UCP4 and UCP5 are widely involved in neuroprotective and antioxidant effects, and play positive roles in the diagnosis and treatment of Parkinson's disease and Alzheimer's disease 13, 14. With the in-depth progress of a series of studies, UCPs are expected to become innovative targets for the treatment of metabolic diseases, providing novel ideas for clinical applications 15.

## Subtypes and structure of UCPs

**Figure 2:**
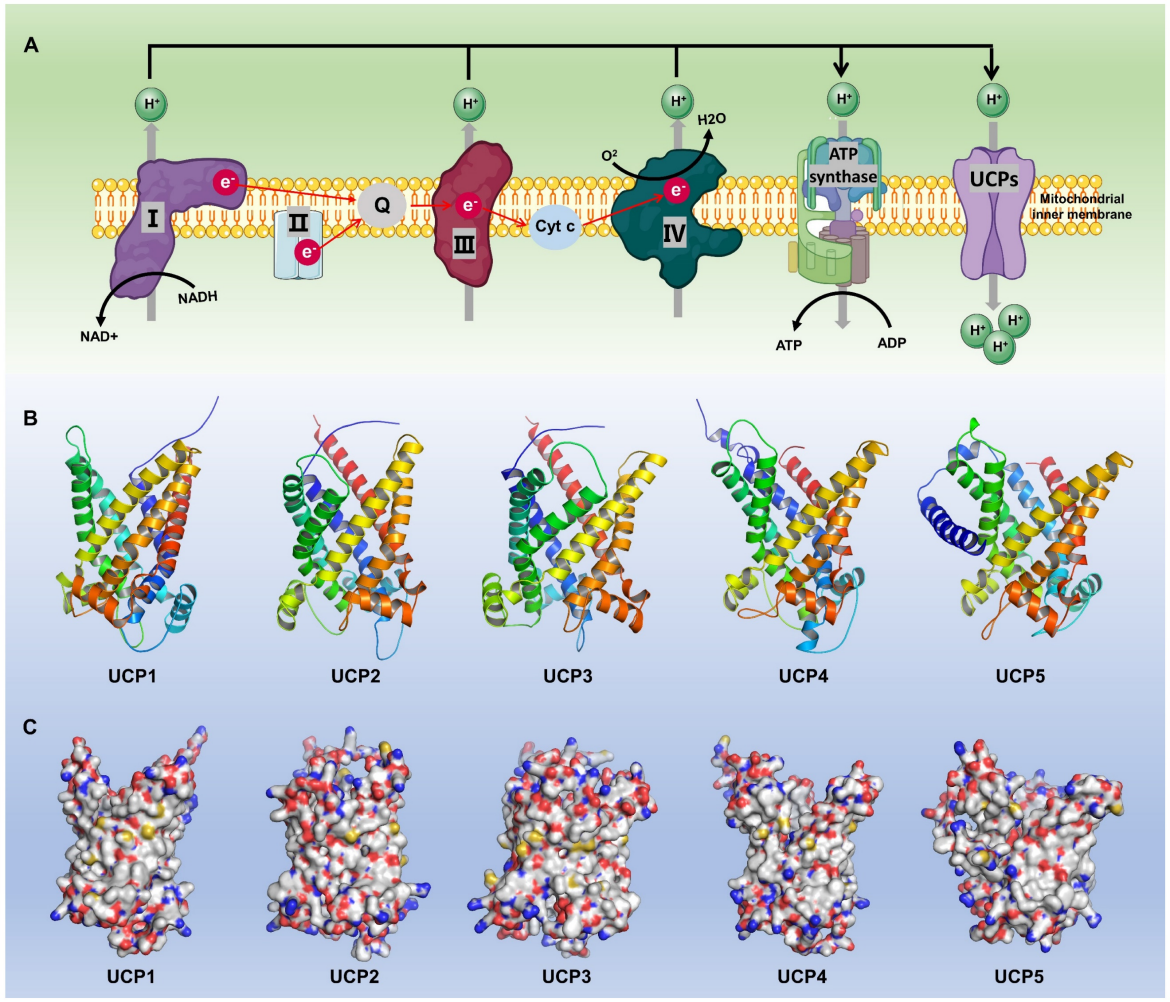
** Basic biological functions and molecular structures of different subtypes of UCPs. (A)** The mitochondrial respiratory chain (I-IV) pumps H^+^ into the mitochondrial intermembrane space, creating a concentration gradient. This electron transfer generates an electromotive force that drives ATP synthase to produce ATP. UCPs, anchored to the inner mitochondrial membrane, are activated by the H^+^ gradient, exerting a range of essential biological functions, including heat production. **(B-C)** Schematic diagrams of predicted protein structures for different members of the UCP family. UCP protein structures were predicted using AlphaFold3 and visualized using Pymol. NAD^+^, Nicotinamide adenine dinucleotide; NADH, Nicotinamide adenine dinucleotide; Cyt C, cytochrome complex.

The expression patterns of UCPs and the structural characteristics of their subtypes are specific in different tissues and physiological states 16** (Fig. 2)**. Current studies have revealed at least five known uncoupling protein subtypes: UCP1, UCP2, UCP3, UCP4 and UCP5, whose names and functions differ slightly across species 17. The subtypes, distribution and functions of each group of UCPs are detailed in **Table 1**. All UCPs are mitochondrial inner membrane transport proteins with some common structural features. The transmembrane structure of UCP is composed of 6 α-helical transmembrane helix domains (TM1-TM6), forming a central proton channel, with both the N-terminus and the C-terminus facing the mitochondrial matrix side, which is conducive to interactions with protons and other molecules 2, 18. UCPs have some functional binding domains such as the purine nucleotide binding domain located on the matrix side, which inhibits proton leakage by binding to ADP/GDP; the fatty acid binding site changes the protein conformation by binding to long-chain fatty acids such as palmitic acid to open the proton channel; cysteine residues are involved in redox regulation, and related studies have shown that Cys305 of UCP3 is easily modified by reactive oxygen species (ROS) 19. In addition, UCP1 has approximately 60% homology with other subtypes, while the homology between UCP2-UCP5 is higher, reaching 70% 20.

UCP1 is a classic member of the uncoupling protein family. It uncouples the mitochondrial proton gradient, preventing protons from passing through the ATP synthase complex to produce ATP, thereby exerting a thermogenic effect and increasing energy consumption 21. UCP1 is distributed mainly in BAT and in the interscapular fat of infants. BAT contributes to heat generation and body temperature regulation through UCP1-mediated non-shivering thermogenesis. UCP1 is distributed in small amounts in WAT and skeletal muscle, but UCP1 expression in skeletal muscle is upregulated under cold adaptation conditions 22. UCP2 is widely distributed in white adipose tissue, liver, spleen, macrophages, pancreatic β cells, heart, kidney, lung, and central nervous system. UCP2 is expressed in almost all tissues, and its expression is particularly significant in immune cells, the nervous system, and some endocrine glands 23, 24. Current research largely suggests that the primary physiological roles of UCP2 and UCP3 are not thermogenic uncoupling like that of UCP1, but rather crucial metabolic adaptation and signal transduction proteins. Unlike UCP1, UCP2 is involved mainly in regulating the oxidative stress response and glucose metabolism 25. Studies have confirmed that UCP2 is closely related to anti-inflammation, antioxidation and cell survival 26. UCP3 is distributed mainly in skeletal muscle, heart and BAT, but is also expressed at low levels in WAT, placenta tissue and breast tissue. UCP3 is extremely important for fatty acid metabolism; and helps maintain energy balance by reducing heat production during fatty acid oxidation. Furthermore, the uncoupling effect of UCP3 is closely related to the metabolic function of muscle. It reduces ROS through mild uncoupling and protects muscle mitochondria from oxidative damage. UCP4 is specifically distributed in the central nervous system, especially in neuron-rich areas such as the substantia nigra, hippocampus, and cortex 27. UCP5 is expressed mainly in brain neurons and glial cells, and in the testes, ovaries, and retina. UCP4 and UCP5 are primarily involved in the regulation of energy metabolism and neuroprotection of the nervous system, and they maintain the mitochondrial membrane potential of neurons, reduce oxidative stress damage, and delay neurodegenerative diseases 28, 29.

Different subtypes of the uncoupling protein family participate in cellular energy metabolism, oxidative stress response, and temperature regulation through their unique structural characteristics and regulatory mechanisms 30. In-depth exploration of the location, structure, and function of UCPs is highly important for understanding the mechanisms of energy metabolism, obesity, diabetes, and other metabolic diseases.

## Biological functions of UCPs in cellular homeostasis

With its diverse subtype distribution and functional differentiation, the UCP family is indispensable for thermogenesis, energy metabolism, oxidative stress, and inflammatory activation 31, 32. Although the core mechanism involves proton leakage, the specific physiological role of each member needs to be further elucidated in combination with tissue-specific and environmental signals 33. Next, we discuss the current studies of UCPs in terms of their physiological mechanisms.

### Maintaining thermogenesis and thermoregulation

**Figure 3:**
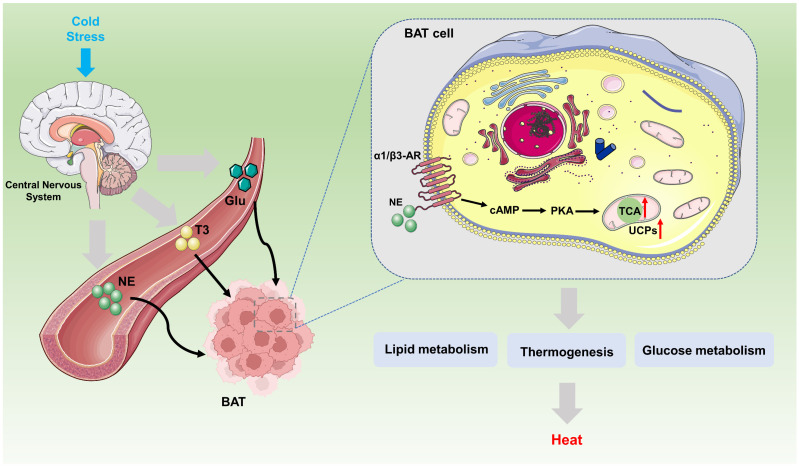
** Biological mechanism pathway of UCPs regulating BAT thermogenesis.** Under external stress stimuli caused by various factors, the sympathetic nervous system, under the complex regulation of the brain's central nervous system, activates the sympathetic nerves, releasing or generating NE, T3, and Glu. These molecules bind to receptors on the BAT cell membrane, such as the α1/β3-AR, activating the downstream cAMP-PKA-UCPs signaling pathway. Specifically, UCPs exert their fundamental biological function of heat production by integrating multiple metabolic pathways, such as lipid metabolism and glucose metabolism. BAT, brown adipose tissue; NE, norepinephrine; T3, triiodothyronine; Glu, glucose; CAMP, 3'-5' Cyclic adenosine monophosphate; PKA, protein kinase A.

Body temperature regulation is an important physiological process for maintaining the stability of the internal environment. Nonshivering thermogenesis (NST) refers to the process of heat production mediated by mitochondrial uncoupling protein in BAT without relying on muscle contraction 34. It is the main heat production method for long-term adaptation to cold environments 30** (Fig. 3)**.

UCP1 is the core molecule involved in nonshivering thermogenesis, and its activity can be regulated by sympathetic nerves, fatty acids and nucleotides 35. Previous studies have shown that the activity of UCP1 is activated by free fatty acids (FFAs), ROS, and synthetic small molecules (such as 2,4-dinitrophenol (DNP)), and is inhibited by purine nucleotides or dysfunction caused by mutations in the purine binding domain 33. Researchers have conducted detailed structural analyses of human UCP1 in the nucleotide-free state, DNP-bound state, and ATP-bound state, and revealed the structural basis of purine nucleotide inhibition of human UCP1, which is conducive to determining the binding site of the DNP activators and revealing the inhibitory mechanism of purine nucleotides 20, 22. However, the specific molecular mechanism by which UCP1 binds to them has not yet been clarified.

Under cold conditions, postganglionic neurons release norepinephrine, which affects the activity of UCP1 via protein kinase A (PKA) through a variety of molecular mechanisms. Existing research conclusions provide evidence for a direct link between sympathetic nerves and UCP1-mediated adaptive thermogenesis. Mechanistically, AIDA, a C2-domain-containing protein located in the outer membrane of mitochondria, is phosphorylated at S161 by PKA and translocated to the membrane space, where it binds to UCP1 and promotes the cysteine oxidation of UCP1 to activate its uncoupling activity 36. In addition, recent studies have shown that BAT activates the tension actomyosin mechanism after adrenaline stimulation. This mechanism is mediated by the mechanically sensitive transcriptional coactivator yes-associated protein 1/WW domain-containing transcription regulator protein 1 (YAP/TAZ) to regulate the thermogenic capacity of adipocytes 37. Identifying this biomechanical signaling mechanism, which is similar to that involved in muscle tissue, is beneficial for promoting the development of brown and beige adipocytes and maintaining thermogenesis. Notably, demyristoylation of gravin-α by histone deacetylase 11 (HDAC11) inhibition was shown to promote UCP1 expression even in a catecholamine-resistant model of adipocytes in which β-adrenergic receptors(β-ARs) signaling is blocked. These findings confirm the potential for inhibiting HDAC11 to therapeutically alter thermogenesis independent of β-AR stimulation 38.

More notably, recent research has uncovered unique pathways involved in adipose tissue thermogenesis and body temperature regulation through UCPs. Creatine kinase b (CKB) as an effector of UCP1-independent thermogenesis), can regulate the energy consumption of adipocytes under cold stimulation in parallel with UCP1 39. Furthermore, GATA3 interacts with the transcriptional coactivator PGC1-α to increase UCP1 expression and promote energy consumption 40. Zhang et al. demonstrated that inhibition of the PPARγ/long noncoding RNA (lncRNA) axis could increase UCP1-dependent and UCP1-independent thermogenesis 41, 42. Interestingly, a study of two different UCP1 isoforms in mice, revealed that the long form (Ucp1L) was more efficiently translated than the short form (UCP1S), and Chen et al. reasonably posited that the cytoplasmic polyadenylation element binding protein 2 (CPEB2)-activated translation of the long 3'-UTR UCP1 mRNA might be conserved in humans 43. With respect to mitochondria, the mitochondrial calcium uniporter (MCU) recruits UCP1 through MCU regulator (EMRE) to form the MCU-EMRE-UCP1 complex, which functions as a thermogenic uniporter. Studies have suggested that mitochondrial calcium uptake 1 (MICU1) may inhibit the formation of thermogenic uniporters to negatively regulate thermogenesis, which is essential for driving UCP1 expression and regulating metabolic disorders 44. Additionally, data suggest that mitochondrial phosphatidylethanolamine (PE) may serve as a responder to regulate UCP1-dependent thermogenesis, modulating UCP1-dependent proton conductance across the inner mitochondrial membrane (IMM) to modulate thermogenesis 45.

### Mediating metabolism and energy balance

By regulating the conversion and utilization of energy, UCPs influence the metabolism of lipids, glucose and other nutrients to ensure that the body adapts to different metabolic needs and maintains energy balance, weight control and metabolic health. Here we summarize the latest research progress on the roles of UCPs in mediating substance metabolism and energy balance.

#### Lipid metabolism

Previous studies have confirmed that UCPs are extremely critical in lipid metabolism, and help maintain energy balance and prevent obesity by promoting lipolysis and fatty acid oxidation, reducing fat storage, improving insulin sensitivity, and regulating adipocyte function 46, 47. Promoting the oxidation of fatty acids is among the core functions of UCPs, especially in BAT 48. By promoting the continued oxidation of fatty acids, allows the body to burn fat and reduce fat storage. In contrast, an obese diet impairs the oxidation of unconjugated substrates and promotes the whitening of BAT 49, 50.

With regard to UCP1, emerging evidence has shown that chronic fatty acid (FA) depletion induces UCP1 expression and that the UCP1 content can compensate for proton-lucent activity under low MMP, further suggesting a regulatory mechanism for UCP1 expression and mitochondrial energy status in human BAT cells under different nutritional conditions 51, 52. Previous studies have confirmed that in UCP1 knockout mice, cold adaptation induced by cold stimulation induces alternative thermogenesis. Hence, the UCP1 knockout mouse model is the most commonly used animal model to determine the antiobesity effect of UCP1, but the actual effect is altered by unknown antiobesity factors in the body 53, 54. Through high-throughput library screening of secreted peptides, two fibroblast growth factors (FGFs), FGF6 and FGF9, have been identified as potent inducers of UCP1 expression in adipocytes and preadipocytes 55. Additionally, research has shown that UCP1 deletion increases the expression of the FGF21 gene in adipose tissue, which is an important source of endogenous FGF21. Endogenous fibroblast growth factor 21 (FGF21) can also serve as a major regulator of protection against diet-induced obesity in the absence of UCP1 56, 57. However, by using UCP1-FGF21 double knockout mice to further analyze the thermogenic effects of UCP1 and FGF21 in cold environments, researchers found that neither UCP1 nor FGF21 is required for maintaining body temperature or metabolic homeostasis in UCP1 knockout mice. These findings imply that more research is needed to better understand the clinical translation of UCP1 and FGF21 and their effects on cardiovascular lipid metabolism 58. Similarly, the paradoxical effects of ambient temperature and UCP1 activation on cardiovascular disease progression have been investigated, with research revealing that thermoneutrality is associated with diminished monocyte egress from bone marrow tissue in a UCP1-dependent manner 59.

Glycerol kinase stimulates UCP1 expression through mediating fatty acid metabolism in beige adipocytes 60. The SIRT5-mediated upregulation of C/EBPβ increases BAT browning through UCP1 61. As a protein phosphatase localized in mitochondria, PGAM family member 5(PGAM5) mediates molecular mechanisms including immunity, apoptosis, and metabolism. Moreover, PGAM5 has been shown to inhibit UCP1 activity and alleviate mitochondrial energy consumption by regulating its phosphatase activity and intramembrane cleavage 62. UCP1 also exhibits diverse expression patterns in different types of adipose tissue and nonadipose tissue 63. The expression of UCP1 in skeletal muscle fiber/adipose progenitor cells is genetically and hormonally controlled 64, 65. Consistent with this, Wu et al. described a host gene‒microbe metabolic axis involving the regulation of intestinal lactate levels and shaping of the gut microbiota via intestinal HIF-2a, which mediates UCP1 and thereby suppresses adipose tissue thermogenesis during obesity 66. Together, these findings reveal the indispensable status of UCP1 in fatty acid metabolism and lipid accumulation.

In addition to the crosstalk between metabolic mechanisms, research on the effects of various exogenous factors on lipid accumulation is also ongoing. Most recently, annonaceous acetogenins mimic AA005, milk fat globule membrane, whey protein and soy protein have been hypothesized to upregulate UCP1 to fight obesity 67-69. Existing data provide evidence that dietary selenium supplementation can enhance the facultative binding of UCP1, providing a potential avenue for reducing obesity 70. Notably, the development of corresponding tools and applications also facilitates the study of the lipid metabolism function of UCPs. For example, exploration of a functional beige adipocyte cell lineage revealed independent cell subclasses expressing UCP1 and ineffective creatine cycling 71.

Except UCP1, other UCPs can impact the activity of enzymes related to fatty acid mobilization and lipolysis. UCP2 enhances the release and mobilization of fatty acids by changing the 5' AMP-activated protein kinase (AMPK) signaling pathway 72, 73. UCP3 was first discovered in 1997, and was shown to catalyze the exchange of C4 metabolites in a manner similar to UCP2 74. Similarly, emerging findings indicate that inhibiting UCP3 can affect the aggregation of mitochondrial supramolecular proteins in mouse BAT, thereby disrupting the fatty acid oxidation process 75.

#### Glucose metabolism and insulin resistance

The ability of UCPs to regulate metabolism is also reflected in glycolysis and insulin sensitivity, and UCPs may have a positive impact on preventing and alleviating obesity and diabetes associated with metabolic syndrome 76. As with body thermogenesis, elevated UCP1 levels also facilitate glucose uptake in human white adipocytes 77. As recently reported, the liver mediates preadipocyte proliferation and UCP1 expression to improve glucose metabolism 78. Catecholamines simultaneously regulate UCP1 by mediating glycogen metabolism. Moreover, the synthesis and degradation of glycogen increase under the action of catecholamines, and glycogen metabolism is beneficial to the body's production of ROS, which leads to p38 MAPK activation, thereby driving the expression of UCP1. These results emphasize that glycogen acts as an indispensable bridge in adipocytes, linking glucose metabolism to thermogenesis 79.

In addition, the interaction between glucose metabolism and thermogenesis is being explored, and BAT ATP-citrate lyase protects against metabolic stress, tightly integrating carbohydrate utilization to thermogenesis, and providing fundamental insights into the fatty acid synthesis-oxidation paradox in BAT 80. In addition, a prior study revealed that TUG cleavage pathway modulates both Insulin-stimulated glucose uptake in muscle and energy expenditure at the biological level by upregulating the expression of sarcolipin and UCP1. Hence, targeting this mechanism would be beneficial in promoting the thermic effect of food, and inhibiting it could lead to obesity 81.

In contrast, emerging studies have identified novel roles that are not linked to UCPs 82. For example, WAT browning or hepatic gluconeogenesis is associated with enhanced glucose clearance when the gut microbiota is absent. However, in a mouse model of gut microbiota depletion, BAT and cecum tissue, but not white adipose tissue (WAT) or liver, contribute to glucose uptake, and this response is independent of adaptive thermogenesis 83, 84. At the cellular level, beige fat changes energy homeostasis through Ca^2+^ cycling. Specifically, Ca^2+^ cycling stimulated by the activation α1/β3-adrenergic receptors or the SERCA2b-RyR2 pathway accelerates UCP1-independent thermogenesis. This atypical thermogenic mechanism, it demonstrated that beige fat can improve glucose tolerance independent of body weight 30. Consistent with this effect, another study revealed that eicosapentaenoic acid profoundly alleviates weight gain and glucose intolerance in UCP1-KO mice via increased oxygen consumption and BAT PGC1α activity, independent of UCP1 85. Nevertheless, our understanding of metabolic and thermogenesis processes *in vivo*, especially in the context of obesity, remains limited, and the effects and relationships between glucose metabolism and UCPs remain to be fully explored.

### Modulating redox homeostasis

**Figure 4:**
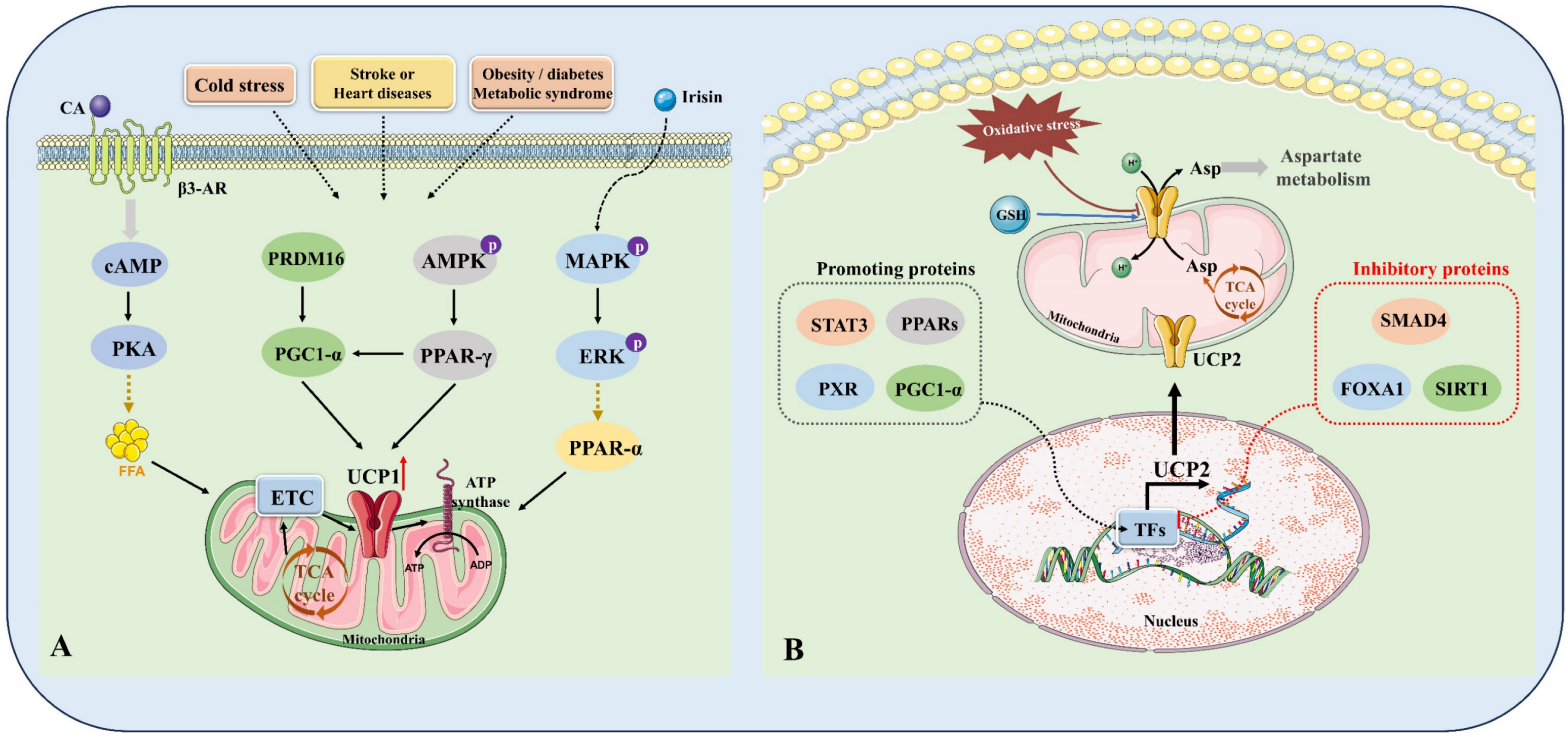
** Intracellular regulatory mechanisms of the key components of UCPs. (A)** Mechanisms of regulating UCP1. In the complex pathophysiological context of cold stimulation, stroke or heart disease, and obesity/diabetes, the body can release catecholamines (CA) through (1) sympathetic activation, which binds to β3-AR on the cell membrane and activates cAMP-PKA-FFA-UCP1 signaling; (2) irisin activates MAPK-ERK-PPAR-α signaling; and (3) intracellular PRDM16-PGC1-α and AMPK-PPAR-γ signaling crosstalk and regulate each other. The above signals together constitute a complex regulatory mechanism network of UCP1. **(B)** Mechanisms of regulating UCP2. (1) STAT3, PPARs, PXR, PGC1-α, etc. act as positive regulators of UCP2; (2) SMAD4 and FOXA1/SIRT1 act as negative regulators of UCP2. Together, these proteins constitute the regulatory network of UCP2, controlling the transcription and translation levels of UCP2, affecting the abundance of UCP2 on the mitochondrial membrane, and thereby regulating metabolic reprogramming, including aspartate metabolism, GSH metabolism, and TCA energy metabolism. FFA, free fatty acid; MAPK, mitogen-activated protein kinase; AMPK, adenosine monophosphate activated protein kinase; ERK, extracellular signal regulated kinases; PPAR-γ, peroxisome proliferator-activated receptor-γ; TFs, transcription factors; STAT3, signal transducer and activator of transcription 3; PPARs, peroxisome proliferator-activated receptors; PXR, pregnane X receptor; PGC1-α, peroxisome proliferator-activated receptor γ coactivator 1 alpha; SMAD4, SMAD family member 4; FOXA1, forkhead-box protein A1; SIRT1, NAD-dependent deacetylase sirtuin-1; GSH, Glutathione.

UCPs significantly decrease ROS production by lowering mitochondrial membrane potential, minimizing electron leakage in the electron transport chain, and controlling calcium ion homeostasis, thereby alleviating oxidative stress. As indispensable regulators of oxidative stress, UCPs play important protective roles in various metabolic diseases and ageing 21, 86. Now we summarize the evidence supporting the leading roles of UCPs in oxidative stress **(Fig. 4)**.

#### The role of UCPs in mitochondria

Although the mitochondrial membrane potential (ΔΨm) is essential for ATP synthesis, it also increases the probability of electron leakage in the electron transport chain, leading to increased ROS formation 87. UCPs reduce membrane potential through proton leakage, decrease electron leakage, and inhibit ROS generation. Furthermore, complexes I and III of the electron transport chain are the main sites of ROS generation 88. UCPs also decrease the accumulation of electrons in these complexes by lowering the membrane potential, thereby reducing the content of superoxide. By constructing an *in vivo* mouse AKI model and an *in vitro* human renal proximal tubular epithelial cell aristolochic acid I model, documented studies have confirmed that UCP1 and UCP2 respond positively to the protective effect of kidney injury by fighting oxidative stress 11, 89. Combined with the results of skeletal muscle proteomic analysis, these findings revealed that compared with wild-type controls, mice ectopically expressing UCP1 in skeletal muscle presented lower systemic methylglyoxal-derived advanced glycation end product exposure flux, representing better aging performance, extended lifespan, and greater resistance to impaired metabolic health 90. Similarly, it was recently reported that UCP2 alleviates mitochondrial dysfunction and oxidative damage in substantia nigra pars reticulata with hepatic encephalopathy 91. In contrast to the above results, Sunad Rangarajan et al. have shown that silencing UCP2 *in vivo* induced regression of fibrosis in an aged mouse model of pulmonary fibrosis, providing evidence for the therapeutic targeting of UCP2 in age-associated organ fibrosis and impaired tissue regeneration 92. These up-to-date studies illustrate that, UCPs also maintain Ca^2+^ homeostasis and decrease ROS production by indirectly affecting the uptake and release of Ca^2+^, thereby further protecting the integrity of mitochondrial structure and function. Aldolase B (ALDB), a glycolytic enzyme, is related to decreased calcium release from the endoplasmic reticulum (ER). It was recently discovered that UCP2 and ALDB influence insulin secretion by modulating mitochondrial activity and endoplasmic reticulum Ca^2+^ release, suggesting that targeting the UCP2/ALDB axis is a promising approach for restoring β-cell function 93.

With respect to the molecular mechanisms of oxidative stress, the expression and activity of UCPs are guided by multiple signaling pathways, and these processes in turn act on these patterns. As previously stated, UCPs mediate energy metabolism and ROS formation by influencing AMPK activity, regulating energy balance and antioxidant capacity. Moreover, UCPs expression is dominated by the expression of transcription factors such as PPARs, and CREB and epigenetic modifications. Previous studies have confirmed that ER stress diminishes UCP1 expression by restraining PPARγ binding activity in mouse beige adipocytes, and that procyanidin B2 targets and activates PPARγ/UCP1 signaling, exacerbating oxidative stress and thus subtly improving the developmental capacity of bovine oocytes 94, 95.

#### The role of UCPs in oxidative stress-related signaling pathways

To explore the relationships between UCPs and other transcription factors, Nrf2, a major transcription factor involved in the antioxidant response, was used to regulate various antioxidant enzymes, including superoxide dismutase and glutathione peroxidase. UCPs further promote antioxidant defense by reducing ROS levels and enhancing the activity of Nrf2. In addition, UCPs lower ROS generation and inhibit overactivation of the nuclear factor kappa B (NF-κB) pathway, ultimately reducing inflammatory responses and apoptosis. MAS, as a G protein-coupled receptor, is considered a new component of the renal angiotensin system (RAS). In a rat model of early brain injury in subarachnoid hemorrhage (SAH), AVE 0991, a selective agonist of Mas, has been reported to alleviate oxidative stress and neuronal apoptosis through the MAS/PKA/CREB/UCP2 pathway, which demonstrates that AVE may be a novel and effective therapeutic approach against oxidative stress in SAH patients 96. Another study highlighted the role of UCP4 in mitochondrial homeostasis, clarifying the role of UCP4 in intermittent hypobaric hypoxia (IHH). Specifically, UCP4 overexpression alleviated oxidative stress damage in cerebellar Purkinje cells of IHH mice, enhanced mitochondrial function, and improved IHH-induced movement disorders 97.

Considering the relevance of UCPs in the biological process of oxidative stress, several novel medicines and clinical applications that rely on UCPs have been discovered. Both *in vitro* and *in vivo* experiments have shown that, dietary fish oil supplementation ameliorates adipose tissue dysfunction, oxidative stress, PPARγ, and UCP2 in insulin-resistant rats 98, 99.

### Regulating the inflammatory response

There is a complex bidirectional regulatory relationship between UCPs and the inflammatory response. UCPs regulate mitochondrial function and the redox state to affect the inflammatory response, and the inflammatory response affects the expression and activity of UCPs through cytokines and signaling pathways 100. Following that, we detail the most recent interaction mechanism between these factors.

#### UCPs and inflammatory cells

During the activation of inflammation, macrophages, neutrophils, and lymphocytes rapidly recruit and release a large number of inflammatory factors. At the cellular level, high expression of UCP2 decreases macrophage polarization towards a pro-inflammatory phenotype (M1 type). Furthermore, macrophages switch from oxidative phosphorylation to glycolysis under stimulation caused by inflammation, thereby regulating UCP2 through AMPK and mTOR signaling. While in T cells, UCP2 affects mitochondrial metabolism and the differentiation and functionality of T cells, ultimately affecting immune response strength. However, a recent study reported that UCP2 activity in adipose tissue during obesity-induced insulin resistance is not critical for macrophage activation 101. In addition to this discovery, another type of immune cell, mast cells, is enriched with tryptophan hydroxylase 1 (Tph1), which is the rate-limiting enzyme for peripheral serotonin synthesis. Yabut et al. reported that Tph1 deletion in mast cells enhances UCP1 expression in BAT, further limiting the development of obesity and insulin resistance, and providing insights into unique avenues for treating obesity and diabetes 102.

Notably, inflammation also alters the methylation status of the promoter region of UCP2 and subsequently affects its transcriptional activity. Similarly, another finding revealed that the cysteine 253 residue of UCP1 is involved in sex-dependent inflammation in adipose tissue 103. Histone deacetylase 3 (HDAC3), a transcriptional coregulator, whose activity requires interaction with nuclear receptor corepressors (NCoRs) and is closely related to the thermogenic function of UCP1. Interestingly, in the absence of HDAC3 activity, BAT lacking NCoRs activates inflammatory pathways that stimulate BAT through the sympathetic nervous system, resulting in normal heat production. This discovery provides evidence for our in-depth elucidation of the multiple physiological effects of the transcription core complex 104. In summary, all these results emphasize that inflammation-mediated metabolic reprogramming and epigenetic regulation are also involved in influencing UCPs.

#### UCPs and inflammatory factors

NF-κB is a core factor in inflammation, and UCP2 has a major role in the anti-inflammatory response. As mentioned in the previous section, ROS, as crucial triggers of inflammation, can activate proinflammatory transcription factors such as NF-κB and AP-1. According to previous findings, UCPs decrease ROS levels and restrain the activation of the above transcription factors, resulting in low expression of proinflammatory cytokines, including TNF-α, IL-6, and IL-1β or fibrogenesis TGF-β 105. In terms of the potential underlying mechanisms, several previous studies have established that UCP2 inhibits NF-κB through weakening ROS levels and increasing mitochondrial function 106. For example, Aspergillus protease-induced mitochondrial ROS generation is associated with the downregulation of UCP2 expression through the TGF-β-SMAD4 signaling pathway, which further clarifies the mechanism of bronchial epithelial inflammation caused by allergic fungal proteases 107. In addition, UCP2 overexpression suppresses the nuclear translocation of NF-κB, reduces the transcription of downstream proinflammatory genes, and alleviates inflammatory diseases 108.

Additionally, in both UCP1 knockout mouse and pig models, Ping Gu et al. found that UCP1 deletion promotes increased mitochondrial membrane potential and mitochondrial superoxide production, which contributes to overactivation of the NLRP3 and caspase1-mediated maturation of IL-1, and highlighted that the vascular protective effect of UCP1 is partly due to the anti-inflammatory effect of UCP1 109. These data shed light on the role of UCP1 in maintaining vascular health in perivascular adipose tissue. In contrast to these representative inflammatory factors, other inflammatory factors, such as IL-33, have been previously shown to trigger the early expression of proinflammatory genes in macrophages and subsequent differentiation into alternatively activated macrophages (AAMs). However, the molecular mechanisms controlling this differentiation have not been clearly elucidated. A recent study revealed that UCP2-mediated mitochondrial reprogramming promotes IL-33-induced AAM differentiation 110. Inflammation is generally accompanied by apoptosis. In line with the findings of previous study, UCPs also inhibit apoptosis by restraining the opening of mitochondrial permeability transition pores (mPTPs), thus attenuating inflammation-induced tissue damage.

## Research progress on UCPs in diseases

**Figure 5:**
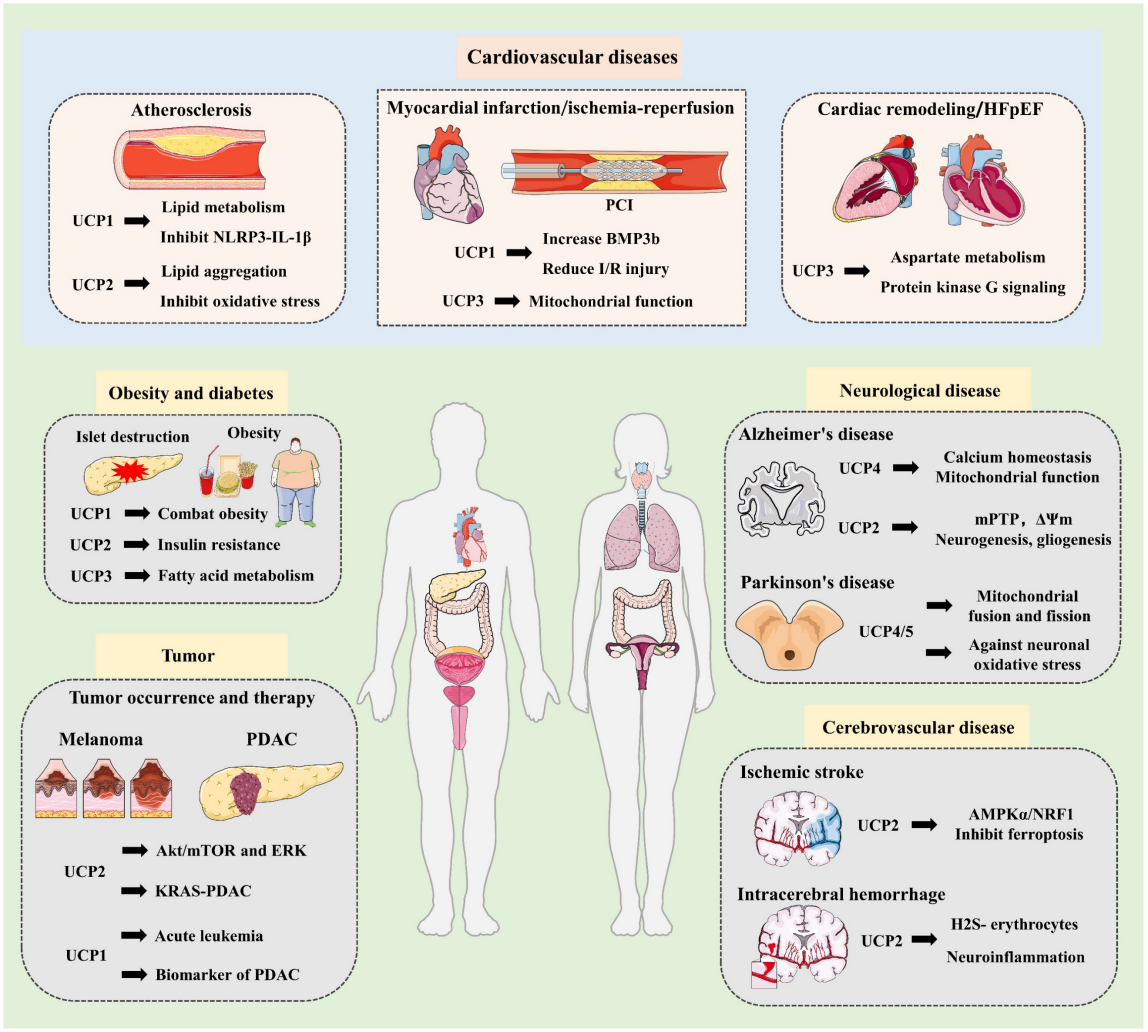
** Research progress of UCPs in multisystem diseases.** UCPs are involved in the development and progression of various metabolic diseases, including obesity and diabetes, cardiovascular disease, neurological diseases, and cancer. UCP1-3 combats obesity and diabetes by improving insulin resistance and fatty acid metabolism. In cardiovascular disease, UCP1 improves atherosclerosis by mediating lipid metabolism and inhibiting the NLRP3-LI-1β inflammatory pathway. Similarly, UCP2 inhibits atherosclerosis by reducing lipid accumulation and oxidative stress. In myocardial infarction and ischemia-reperfusion injury, UCP1 promotes the cardioprotective factor BMP3b to reduce IR, while UCP3 improves mitochondrial function to exert vascular protection. Furthermore, UCP3 mediates aspartate metabolism and regulates protein kinase G signaling to alleviate cardiac hypertrophy and HFpEF. In neurological diseases, UCPs target calcium homeostasis and mitochondrial function to alleviate neurodegenerative diseases, including Alzheimer's and Parkinson's disease. UCP2 inhibits ferroptosis and inflammatory responses, effectively improving neurovascular disease. Additionally, UCPs are widely involved in tumorigenesis and progression. UCP2 promotes melanoma through Akt/mTOR and ERK and also mediates KRAS to affect PDAC. Furthermore, studies have found that UCP1-mediated thermogenesis is associated with acute leukemia, and increased UCP1 expression can serve as a biomarker for early-stage PDAC. These findings strongly suggest that UCPs-guided targeted therapies or multi-target combination therapies will become innovative treatments in the future. NLRP3, NOD-like receptor protein 3 inflammasome; PCI, percutaneous coronary intervention; HFpEF, heart failure with preserved ejection fraction; mPTP, mitochondrial permeability transition pore; AMPKα, AMP-activated protein kinase alpha; NRF1, nuclear factor E2-related factor 1; PDAC, pancreatic ductal adenocarcinoma; mTOR, mammalian target of rapamycin; ERK, Extracellular signal regulated kinases; KRAS, Kirsten rat sarcoma viral oncogene

UCPs are involved in the regulation of cellular homeostasis and metabolic diseases through their diverse molecular structures and functions 71, 111. Specifically, UCPs influence the occurrence and development of obesity, diabetes, cardiovascular diseases, neurodegenerative diseases and related inflammatory diseases 112, 113. More importantly, UCPs exert anticancer effects by reprogramming carcinoma metabolism and improving drug resistance, and provide strategies for delaying ageing through antioxidant damage** (Fig. 5)**.

### Obesity and diabetes

In line with what is described in the above section, most of the UCPs research to date has been in the field of weight loss. Mechanistically, UCPs regulate the efficiency of energy metabolism and alter its activity to affect fat storage and burning. UCP1, UCP2, and UCP3 have considerable implications in obesity, diabetes, and metabolic syndrome 114-116. In particular, the expression of UCP1 in BAT is closely related to thermogenic function, increasing energy expenditure to combat weight gain. Therefore, researchers are actively exploring therapeutic strategies to activate UCP1 to combat obesity 30, 36. Contrary to the commonly held assumption, it was also discovered that increased UCP2 expression is often strongly associated with diabetes occurrence and insulin resistance 117. Although UCP2 protects islet β cells from oxidative stress to some extent by reducing intracellular ROS levels, excessive UCP2 activity triggers ROS outbursts and impairs islet β cells and insulin secretion 106, 118. In other words, UCP2 is widely expressed in the pancreas, liver, muscle, and adipose tissue. The active regulation of pancreatic islet β cells, has been reported to cause metabolic dysfunction, decrease ATP synthesis, and restrain insulin secretion through a reduced ATP/ADP ratio 119, 120. In addition, in liver and adipose tissue, abnormal UCP2 content may lead to fatty acid metabolism disorders, which in turn exacerbate insulin activity 119. However, similar to UCP1, UCP3 mainly enhances fatty acid oxidation and reduces fat accumulation to coordinate energy balance 119. UCP3 overexpression has also been shown to help reduce fat accumulation and improve metabolic status, and UCP3 deficiency also exacerbates fatty acid accumulation, which ultimately contributes to insulin resistance and diabetes progression 121.

Obesity is a major risk factor for type 2 diabetes 122. As outlined above, obesity alters the expression and activity of UCPs, causing a decrease in the efficiency of energy metabolism, thus exacerbating the development of obesity and diabetes 105, 123. With respect to the critical role of UCPs in energy metabolism, modulating UCPs will be an effective strategy for the treatment of obesity and diabetes in the future. Consistent with these findings, metabolic disturbances in diabetes and obesity can be ameliorated by increasing UCP1 activity, activating thermogenic processes, or inhibiting UCP2 overexpression 24, 124. The subsequent challenge is to clarify their various site-specific downstream targets and determine their unique signaling roles, further providing evidence for the clinical translation of UCPs.

### Cardiovascular diseases

Cardiovascular disease (CVD) is the main threat to human health. Several studies have shown that UCPs participate in the development of various cardiovascular diseases by regulating energy metabolism, oxidative stress, the inflammatory response, apoptosis and other molecular pathways 10. The expression patterns and signaling mechanisms of UCPs differ in response to different types of CVD. Herein, we provide an overview of the most recent findings concerning the role of UCPs in various forms of CVD (**Table 2**).

#### Hypertension

Hypertension is an important indicator of a range of cardiovascular diseases, and its pathophysiology has attracted considerable attention. Previous studies have shown that UCPs can impact vascular tone and blood pressure stability by mediating the mitochondrial activity of vascular smooth muscle cells.

In an experimental analysis of clinical plasma samples, available data revealed lower circulating UCP2 levels in patients with T2DM, which were associated with endothelium-dependent vasodilation in conduit vessels. Notably, these findings imply that UCP2 can be utilized as a biomarker for endothelium function in T2DM patients, but further research should be conducted to clarify the link between UCP2 alterations and blood glucose levels and other cardiovascular event risks 125. Consistently, in a stroke-prone spontaneously hypertensive rat model, oxidative stress and inflammatory activation were attenuated, and renal injury and stroke risk were decreased 126. Nevertheless, another mouse experimental study examining the association between UCP2 and blood pressure (BP) yielded beneficial results. Specifically, DJ-1, also known as PARK-7, is a multifunctional oxidative stress response protein. When DJ-1 is depleted, oxidative stress induces hypertension, and NO function decreases. These findings confirmed that UCP2 overexpression in the kidney increased the blood pressure of DJ-1 knockout mice, indicating that excessive UCP2 levels may have adverse effects on BP regulation. Taken together, these findings suggest that UCP2 acts as an antioxidant and is involved in intricate mechanisms in hypertension. In terms of the expression level, activation time, and environmental variables, UCP2 can negatively impact physiological processes 127.

#### Atherosclerosis

Atherosclerosis (AS) is a chronic inflammatory disease involving pathological processes such as lipid deposition, smooth muscle proliferation, fibrosis and calcification. The initiation of atherosclerosis is usually multifactorial and involves the induction of endothelial cell damage. In human aortic and human umbilical vein ECs, Krüppel-like factor 2 (KLF2) has been shown to mediate fluid shear stress-dependent regulation of UCP2 expression. Both *in vitro* and *in vivo* experiments have shown that, UCP2 is a novel mechanosensitive gene in ECs that is controlled by fluid shear stress and KLF2. Moreover, RNA-seq analysis revealed that, Forkhead box protein O1 (FOXO1) is a major proinflammatory transcriptional regulator activated by UCP2 knockdown, and that the inhibition of FOXO1 induced by UCP2 expression is closely associated with the phosphorylation of AMPK. Therefore, it has become increasingly evident that UCP2 expression is critical for endothelial proinflammatory responses and atherosclerosis 128. Apart from this, UCP2 is also found to be associated with plaque stability, especially with respect to macrophage mitochondrial function. According to existing research results, UCP2 prevents atherosclerosis by lowering lipid buildup and suppressing oxidative stress. In contrast, some studies suggest that excessive UCP2 activity can induce mitochondrial dysfunction and heart disease. Consequently, the link between UCP2 and AS is relatively complex and the function of endothelial UCP2 signaling in plaque rupture remains to be determined.

Lipid accumulation is a critical element in the process of atherosclerosis. Focusing on other UCPs, UCP1-mediated thermogenesis can alleviate lipid accumulation and offers extensive applicability for the treatment of AS. For instance, C-type natriuretic peptide (CNP) is a multidimensional protective factor in the cardiovascular system that impacts local blood flow, angiogenesis, and cardiac function. In CNP-knockout mice, researchers have reported that the thermogenic phenotype correlates with increased UCP1 levels and preferential mitochondrial utilization of lipids, implying that UCP1 regulates lipid metabolism to maintain vascular health 129. As a consequence, an increasing number of analyses are looking into novel strategies to treat AS aiming at UCP1. AP39, a mitochondrial hydrogen sulfide donor, has been proven to stabilize atherosclerotic lesions by increasing UCP1 expression in vascular smooth muscle cells, lowering overall macrophage concentration and collagen deposition 130. Furthermore, using UCP1 as a target, phenolic acid compounds such as ferulic acid and protocatechuic acid have been shown to have therapeutic benefits for AS through the inhibition of the NLRP3-IL-1β inflammatory pathway. This presents a theoretical foundation for the precise use of phenolic acid-rich food resources 131.

#### Myocardial infarction and ischemia-reperfusion injury

Myocardial infarction (MI) is typically caused by thrombosis or the rupture of atherosclerotic plaques. Ischemia-reperfusion (IR) injury is a serious consequence of MI that worsens myocardial damage, extends recovery time, and increases patient mortality and complication risk. Prior studies on the underlying molecular mechanism, revealed that UCP3 is expressed primarily in skeletal muscle and the heart; hence, it plays a significant component in the pathophysiology and prognosis of MI and IR. By suppressing the opening of mitochondrial permeability transition pores to sustain mitochondrial function through interactions with adenine nucleotide translocator and the PI3K/AKT pathway, UCP3 has been shown to eliminate the processes of cardiomyocyte apoptosis and necrosis and ultimately improve the prognosis of MI 132. In this context, patients with insulin resistance and T2DM suffer worse cardiac endpoints following MI. Using Sprague-Dawley rats model with myocardial-specific UCP3 knockout. Edwards et al. have concluded that normal cardiac UCP3 levels are essential for the restoration of long-chain fatty acid oxidation, mitochondrial respiratory ability and myocardial contractility after I/R. Moreover, we contend that matching fatty acid metabolism to the energy needs of the heart, especially through supplementation with medium-chain fatty acids, might be a promising approach to improve IR injury for patients with T2DM heart disease 133. With respect to other UCPs, a recent study evaluated the role of BAT in IR using UCP1-deficient mice, and revealed a cardioprotective protein that is released as bone morphogenetic protein 3b (BMP3b). Notably, BMP3b mediates cardioprotection through SMAD1/5/8, and its plasma levels are correlated with the magnitude of myocardial injury. As a consequence, increasing BMP3b levels or targeting Smad 1/5 may provide theoretical possibilities to ameliorate myocardial damage in I/R injury 134.

#### Heart failure

Heart failure is a common complication of several cardiovascular disorders, including cardiomyopathies, myocarditis, congenital heart disease, pericarditis, and endocarditis. Previous research has verified the presence of BAT in adults using noninvasive quantitative measurement with (18)F-FDG PET-CT, emphasizing the necessity of identifying and understanding its composition for human metabolism. A recent case‒control trial quantified the expression level of UCP1 in brown adipose tissue (BAT), and the results confirmed that UCP1 expression in BAT is negatively correlated with cardiovascular metabolic risk in adults, highlighting the potential application of targeting and activating BAT in metabolic diseases 135, 136. In accordance with RNA sequence and STRING bioinformatics analysis, UCP2 suppression is associated with notable changes in the cellular cycle, survival signaling pathways, and mitochondrial genes 137. Although UCP2 inhibition has been found to reduce cardiac dysfunction and heart failure in pressure overload-induced left ventricular hypertrophy, it has a distinct effect on a mouse model of pressure overload-induced right heart failure. More specifically, mouse cardiac fibroblasts express more UCP2 than myocytes do, allowing UCP2-deficient mice to maintain certain cardiac functions under right ventricular pressure overload 138. In the treatment of heart failure with preserved ejection fraction (HFpEF), UCP3 deficiency-mediated mitochondrial oxidative stress exacerbates angiotensin II-induced hypertensive left ventricular diastolic dysfunction and exercise intolerance 139. Moreover, recent research has demonstrated that UCP3 expression is downregulated in hypertrophic hearts and cardiomyocytes, while increasing the overexpression of UCP3 improves cardiac hypertrophy by decreasing aspartate levels 140. Mitochondrial antioxidants have shown favorable effects on HFpEF, although research on their efficacy in animal models and their interactions with UCP3 deficiency is ongoing 139.

Cardiac dysfunction can be caused by a range of heart illnesses, genetic abnormalities, or systemic diseases. Doxorubicin-induced cardiotoxicity can also occur with heart failure and should not be overlooked during cancer chemotherapy. Current research confirms that UCP acts as a target molecule and signal bridge in various types of doxorubicin-induced cardiotoxicity therapies. For example, Irisin has been shown to alleviate DOX-induced pericardial fibrosis via decreasing ROS accumulation, autophagy impairment, NF-κB pathway activation, and the ENDMT phenotype primarily targeting UCP2. Notably, these findings provide a preliminary investigation of the potential of irisin as a cardiokine, but its regulatory mode and mechanism in the cardiac microenvironment remain to be clarified 141. Moreover, Matrine, an alkaloid with antioxidant and anti-inflammatory properties, can also alleviate oxidative stress and cardiomyocyte apoptosis in DOX-induced cardiotoxicity via the AMPKα/UCP2 pathway 142 Further exploration of the specific site mechanisms by which traditional Chinese medicine components and known Western medicines mediate the improvement of cardiovascular diseases by UCPs may lead to more precise treatment through drug combination therapies and the development of target inhibitors or agonists.

### Neurological diseases

The physiological functions of UCPs in neurological illnesses extend beyond directing mitochondrial function and oxidative stress. UCPs play a critical role in neuroprotection through numerous processes, including modulating metabolism, inflammation, and neuroplasticity. We review the latest developments in the use of UCPs in the treatment of neurodegenerative illnesses, cerebral ischemia and reperfusion injury, epilepsy, and other neuroinflammatory diseases.

#### Neurodegenerative diseases

Neurodegenerative diseases are characterized by the ongoing degradation of neuronal structure and function. The main pathogenic features are neuronal death, synaptic loss, and aberrant protein aggregation. Alzheimer's disease (AD), Parkinson's disease (PD) and Huntington's disease (HD) are among the most prevalent neurodegenerative diseases. Among UCPs, UCP2, UCP4, and UCP5 are highly expressed in the central nervous system and are associated with neuronal energy metabolism and antioxidant defense.

AD is characterized by the formation of β-amyloid protein (Aβ) plaques and neurofibrillary tangles (NFTs) triggered by abnormal Tau protein phosphorylation 143. AD patients frequently experience mitochondrial dysfunction 144. As demonstrated in previous studies, UCP4 maintains calcium ion homeostasis and mitochondrial membrane potential to protect neurons from Aβ injury, and utilizes mitophagy to eliminate damaged mitochondria, which hampers the progression of AD 145. Similarly, UCP2 modulates the opening of the mitochondrial permeability transition pore (mPTP), suppressing cell death and substantially reducing the decrease in mitochondrial membrane potential and ROS production triggered by Aβ 146. More crucially, UCP2 and UCP4 gene polymorphisms can increase the risk of AD 147, 148. Recent findings revealed that endothelial UCP2 deficiency eliminates vascular diameter and affects the transition time between neurogenesis and gliogenesis 149. These results suggest that future research should concentrate on expanding the applications of UCPs in the early detection of AD, and evaluating their potential as biomarkers, with the goal of developing AD treatment regimens that target UCPs.

In contrast to AD, which occurs mostly in the hippocampus and cortex, PD largely occurs in the substantia nigra and striatum 150. Mechanistically, AD is caused by the loss of dopaminergic neurons, leading to motor delay or anxiety, depression, sleep disorders, etc. 151. However, in terms of neuronal protection, UCPs particularly UCP4 and UCP5 limit ROS accumulation in dopaminergic neurons by affecting the mitochondrial membrane potential (MMP), and by regulating mitochondrial fusion and fission to maintain energy balance 13. DJ-1, also known as PARK7, has been implicated in the pathogenesis of PD. Prior research has demonstrated that DJ-1 stimulates UCP4 overexpression to guard against neuronal oxidative stress damage by mediating autooxidation and the NF-κB pathway 152. In addition, a study of hepatic encephalopathy-induced basal ganglia motor neuron damage, revealed that UCP2 plays a positive antioxidant role. Mechanistically, this effect is achieved by affecting the levels of K^+^-Cl^-^ cotransporter-2 on GABAergic neurons, which also indicates the considerable potential of the neuroprotective effect of UCP2 in the treatment of movement disorders 153. However, in a mouse glaucoma model, UCP2 knockout promoted mitophagy and reduced retinal ganglion cell death 154. Therefore, these findings suggest that the regulatory effects of UCP2 on different neurological diseases involve multiple pathways and outcomes. In addition, with respect to HD aetiology, UCPs could potentially ameliorate calcium ion signaling, subsequently relieving cellular stress induced by mutant Huntingtin and improving neuronal degeneration in patients 155.

#### Cerebrovascular disease

In cerebral ischemia and reperfusion injury, UCPs may prevent excessive ATP consumption by lowering the mitochondrial membrane potential, and delaying energy depletion during ischemia. As previously stated, UCPs safeguard mitochondrial structure and function by limiting the rapid production of ROS. UCPs can also hinder cytochrome C leakage and initiate of apoptotic cascade processes by blocking the opening of the mPTP. For example, Lei Wang et al. reported that UCP2 expression gradually decreases with prolonged ischemic time in *in vitro* and *in vivo* ischemic stroke models. Specifically, UCP2 activates AMPKα/NRF1 signaling to protect the brain from ischemia-induced ferroptosis 156. Moreover, a study that investigated the influence of UCP2 on the dynamic balance of mitochondrial fission and fusion in ischemic mice under normal and hyperglycaemic conditions revealed that, UCP2 deficiency enhanced ROS generation, early damage to mitochondrial ultrastructure, and disruption of mitochondrial fission dynamic equilibrium, resulting in an increase in the cerebral infarction area 157.

UCP2 is also significantly involved in intracerebral hemorrhage (ICH), and hematoma clearance is primarily accomplished through the phagocytosis of red blood cells in the hemorrhagic brain. Furthermore, spontaneous hematoma clearance after ICH requires mitochondrial complex I and UCP2. Following ICH, the gasotransmitter hydrogen sulfide (H2S), also known as the endogenous modulator of continuous phagocytosis, targets mitochondria and turns on UCP2 to stimulate microglial phagocytosis of erythrocytes 158. Similarly, succinate has been confirmed to activate UCP2, lessen neuroinflammation, and provide protection following cerebral hemorrhage 159.

#### Other neuroinflammatory diseases and complications

According to recent studies, microglial UCP2 mediates inflammation and obesity caused by high-fat diets and supports the essential central regulatory mechanism directed by fuel supply-driven mitochondrial mechanisms 24. In the adult brain, microglia participate in synaptic remodeling through the engulfment of synaptic components, a process whose mechanism of action has yet to be determined. By evaluating microglia‒neuron interactions in the ventral hippocampus, researchers have shown that the conditional ablation of UCP2 in microglia leads to the accumulation of ROS and lysosome‒lipid droplet complexes, which mediate ventral hippocampal circuit function and anxiety-like behaviors 160. Overall, these findings reveal extensive interactions among UCP2, microglia, and neurons, which form a signaling network for anxiety behavior. In comparison to other UCPs, UCP1 plays a role in reducing anxiety-like behaviors, a function independent of its activity in BAT 161. Compared with normal subjects, smokers have higher levels of UCP1 expression in WAT, and studies have shown that nicotine induces browning of WAT through the kappa opioid receptor, which also serves as a channel for modulating brain mechanisms to combat obesity 162.

Nevertheless, UCPs additionally govern energy supply and mitochondrial dynamics, modulating brain network stability and synaptic plasticity, which in turn influences memory and learning. Drosophila melanogaster Uncoupling Protein-4A (UCP4A) is the first biochemically described UCP4 homolog in all species, and is possibly closely related to the manufacture of β-alanine and N-acetylaspartate in the central nervous system 163. In summary, for the purpose of developing more individualized therapies, future research must further elucidate the intricate molecular processes and regulatory networks of UCPs in the neurological system.

### Tumors

Research on UCPs in tumors has focused mainly on their role in energy metabolism, oxidative stress and apoptosis 164. Previous research has found that UCP2 is overexpressed in a variety of tumors and is associated with metabolic reprogramming and chemotherapy resistance, whereas UCP1 may play a role in BAT-related tumors 165. The following section summarizes the current regulatory mechanisms of UCPs and their potential applications in tumor treatment.

#### UCPs-mediated tumorigenesis and development

In tumor research, each subtype of UCPs has distinct modes of action. Recent research has revealed that activating thermogenic adipose tissue can successfully prevent and treat acute leukemia in mice, although UCP1 is expressed at low levels in the majority of cancers. These findings suggest that further study is essential to completely understand its role in tumors involving adipose tissue 166. In contrast to UCP1, UCP2 is overexpressed in a variety of tumors, including lung, breast, and colon cancer. Numerous studies have shown that UCP2 shields tumor cells from oxidative damage and stimulates their survival and growth by eliminating the production of ROS and mitochondrial membrane potential. For instance, UCP2 expression is increased in melanoma cells, stimulating tumor expansion through the activation of the Akt/mTOR and ERK signaling pathways 167. Additionally, elevated UCP2 expression is related to heightened tumor cell resistance to chemotherapeutic medications 168, 169. Considering the complex mechanism of UCP2 in cancer metabolism, it is considered as a marker of tumor malignant potential 170. A whole metabolome analysis of 20 human primary acute myeloid leukemia (AML) cases revealed that ROS increased UCP2 levels and AMPK phosphorylation, upregulated the expression of the glycolytic enzyme 6-phosphofructo-2-kinase/fructose-2,6-bisphosphatase (PFKFB3), and consequently exacerbated AML 171. Similarly, oncogenic KRAS mutations are strongly associated with human pancreatic ductal adenocarcinoma (PDAC) and regulate the redox equilibrium by regulating glutamine metabolism. Recent studies have indicated that UCP2-mediated aspartate transport is essential for the ability of KRAS to regulate glutamine metabolism and support pancreatic cancer progression, suggesting that UCP2 could be a critical metabolic target for treating refractory pancreatic cancer 172. In general, these findings reveal that UCP2 is broadly distributed in numerous organs and affects the evolution of several malignant tumors. In-depth study of the molecular mechanism of UCP2 is critical for future quasi-medical tumor treatment.

With respect to other UCPs, UCP3 is mostly expressed in skeletal muscle and heart, and its role in malignancies has received less attention. However, studies have shown that UCP3 modulates fatty acid oxidation, which affects tumor cell metabolism and survival. Furthermore, UCP4 and UCP5 influence tumor development and apoptosis in nervous system cancers such as gliomas by controlling mitochondrial function and lowering ROS levels. Furthermore, UCP4 and UCP5 levels affect the growth and death of nervous system tumors, including gliomas 173, 174. Comprehensive studies into the specific activities of each UCP subtype in various types of tumors will assist with the development of innovative tumor therapy techniques.

#### Prospects of UCPs in tumor treatment and prognosis

Tumor escape and immunosuppression are challenging issues in tumor treatment. Myeloid-derived suppressor cells in the tumor microenvironment (TEM) reduce uncoupling protein 1 expression, thereby enhancing immunosuppressive activity, emphasizing the critical function of UCP1 in immune evasion 175. The increased expression of UCP1 in abdominal subcutaneous fat could serve as a biomarker of early-stage pancreatic ductal adenocarcinoma, although more in-depth studies need to be conducted 176. Nevertheless, a novel concept known as tumor cell “slimming” provides an emerging treatment strategy for tumor formation. Specifically, phospholipase C-like 1(PLCL1)/UCP1-mediated lipid browning decreases aberrant lipid accumulation, and therefore decreases the progression of clear cell renal cell carcinoma (ccRCC) 177. Consistently, melatonin activated peroxisome proliferator-activated receptor γ coactivator 1A (PGC1A) and UCP1-dependent lipophagy and lipid browning programs, mediating “tumor slimming” to inhibit tumor progression, and revealing the therapeutic potential of monitoring and manipulating UCP1 in ccRCC 178. Undoubtedly, this therapeutic method is based on the fundamental role of UCP1 and expands the new scope of UCP1 application. In another analysis of colon cancer prognosis, UCP1 was discovered to be an independent predictive factor, and its expression was identified as an important prognostic biomarker for colorectal cancer, highlighting that the brown fat-like phenotype is strongly associated to colorectal cancer development 179.

Although UCP2 has been shown to increase the emergence and development of numerous tumors, it also depicts the boundless possibilities of UCP2-targeted treatment options. Beikbaghban et al. reported that UCP2 levels varied dramatically among different types of cancer. Moreover, the abundance of UCP2 is altered by nutritional availability in multiple kinds of cancerous cells, highlighting the complexity and diversity of UCP2 in the regulation of cancer formation 170. With genetic or pharmacological techniques, UCP2-reprogrammed TEM sensitizes melanoma to programmed cell death protein-1 blocking therapy, which leads to beneficial anti-tumor effects 9. Additionally, the UCP2 inhibitor genipin, a natural cross-linker, also exhibits positive antiinflammatory, antitumor and neuroprotective effects and plays an active role in glioblastoma by inducing ferroptosis 180. By restoring oxidative stress equilibrium, disrupting tumor energy metabolism, and enhancing the immune response, UCPs generally show considerable potential for the treatment of malignancies and the prevention of cancer growth. UCP-guided targeted medications or multitarget combination treatment techniques are predicted to emerge as innovative treatments in the future.

## Concluding remarks and prospects

Agonists and inhibitors targeting UCPs have potential therapeutic value in the fields of cardiovascular disease, tumors and other metabolic diseases. The known UCPs regulators are included in **Table 3**. Additionally, through the modulation of UCPs, numerous medicinal compounds and preparations have been shown to be highly effective in preventing metabolic disorders and preserving physiological homeostasis. Current research has indicated that dolutegravir suppresses thermogenesis through modifying the expression of UCP1 in brown/beige adipocytes, contributing to weight gain 181. Similarly, Cyclopia intermedia (Honeybush), Baicalein and Cordycepin have been shown to balance energy expenditure and prevent obesity through UCP1-mediated thermogenesis 182-184. In line with these findings, Yiqi Huoxue granules, a traditional Chinese medicine formula, increase UCP2 levels, reducing hydrogen peroxide-induced apoptosis. TaoHeChengQi decotion improves chronic renal failure by regulating the PHD2/UCP1 and RIPK3/AKT/TGF-β pathways 185. However, to determine their precise regulation and therapeutic application, more studies on the development of medications and reagents related to UCPs are needed.

Owing to the roles of UCPs in a variety of cellular biological processes, many innovative technologies and applications for their study have emerged 186. For example, BML-260, a rhodanine derivative, has been identified as a JSP-1 inhibitor for the treatment of inflammatory and proliferative illnesses related to the dysregulation of JNK signaling. Recently, BML-260 was shown to have thermogenic effects independent of JSP-1 activation of UCP1, which has potential antiobesity implications 187. More importantly, by computationally analysing the molecular structure of AAC and developing a corresponding mathematical model, the researchers found that mitochondrial uncouplers induce proton leakage by activating the ADP/ATP carrier (AAC) and UCP1, thereby paving the way for the development of novel activators that target both 33. Moreover, UCP1 in ThermoMouse was used to track UCP1 expression. Translocator protein-18 kDa (TSPO) is located in the outer mitochondrial membrane, and TSPO-targeting PET can be used to image interscapular brown adipose tissue(iBAT). Cerenkov luminescence imaging of iBAT utilizing a TSPO-targeting PET probe in the UCP1 ThermoMouse could be a more efficient method for reflecting UCP1 expression 188.

In summary, UCP-targeting strategies for metabolic illnesses include gene regulation, medication intervention, physiological stimulation, and lifestyle changes, with the aim of regulating the expression and activity of UCPs to treat diseases such as obesity, diabetes and metabolic syndrome. Recently, intensified exploration in the UCPs field has provided new opportunities to improve cellular homeostasis and health and to further develop precision medicine targeting UCPs.

## Figures and Tables

**Table 1:** Subtypes, molecular characteristics, distribution and functions of UCPs

Subtype	Gene name	Amino acid length	Molecular weight	Main tissue distribution	Subcellular localization	Function
**UCP1**	SLC25A7	~307 aa	≈33.0 kDa	Brown adipose tissue	Mitochondrial inner membrane	Classical thermogenesis, Fatty acid metabolism
**UCP2**	SLC25A8	~309 aa	≈33.2 kDa	Pancreas, immune cells, liver, kidney, brain		Oxidative stress, energy metabolism, immune regulation
**UCP3**	SLC25A9	~312 aa	≈33-34 kDa	Skeletal muscle, heart		Fatty acid metabolism, energy regulation, metabolic adaptation
**UCP4**	SLC25A27	~323 aa	≈36.1 kDa	nervous system		Oxidative stress, neuronal protection, mitochondrial metabolism
**UCP5**	SLC25A14 / BMCP1	~325 aa	≈36.2 kDa	Brain, testicles		Neuronal metabolism, lipid oxidation, mitochondrial function

**Table 2:** Research progress and application prospects of UCPs in cardiovascular diseases

Cardiovascular disease	UCPs	Molecular mechanism	Application Prospects
**Hypertension**	UCP2	mediating the mitochondrial activity of vascular smooth muscle cells	a biomarker for endothelium function
**Atherosclerosis**	UCP1	regulating lipid metabolism, inhibiting the NLRP3-IL-1β inflammatory pathway	a theoretical foundation for the precise use of phenolic acid-rich food resources
	UCP2	reducing lipid aggregation and inhibiting oxidative stress	the function of endothelial UCP2 signaling in plaque rupture remains to be determined
**Myocardial infarction and ischemia-reperfusion**	UCP3	preserving the mitochondrial function, mediating fatty acid oxidation	medium-chain fatty acid supplementation might be a promising approach to improve IR injury for patients with T2DM heart disease
	UCP1	Mediating myocardial protective factor BMP3b	Increasing BMP3b levels or targeting Smad 1/5 could be potential therapeutic options to reducing I/R injury.
**Cardiac remodeling**	UCP3	downregulating the enhanced aspartate	providing a basis for targeting UCP3 to treat cardiac remodeling
**HFpEF**	UCP3	regulating protein kinase G signaling and oxidative stress	the use of antioxidants as an adjuvant therapy for HFpEF
**Doxorubicin-induced cardiotoxicity**	UCP2	alleviating oxidative stress and autophagy, or through the AMPKα/UCP2 pathway	Irisin and Matrine
**Myocardial aging**	UCP2	affecting cellular proliferation and survival	Clarifying UCP2 metabolic properties that influence cardiac function

**Table 3:** Clinical trials of regulators involving UCPs

Name	Identifier	Clinical phase	Conclusion	Reference
**Tirzepatide**	NCT04081337	phase I	increased fat oxidation	189
**Bisoprolol**	NCT04823442	a randomized crossover trial	blunted the mirabegron-stimulated thermogenesis	190
**Rapamycin**	CRAD001ADE12	2-year randomized controlled trial	centrally mediated reduced food intake and increased fat oxidation and mobilization	191
**Boysenberry juice**	UMIN000043476	an open-label single-arm nonrandomized study	increase fat oxidation	192
**High-dose glucocorticoid**	NCT03269747	a randomised, double-blinded cross-over trial	altering skeletal muscle calcium cycling	193
**Glucose-dependent insulinotropic polypeptide**	NCT03734718	a randomized, placebo-controlled, double-blind, crossover study	affects Hepatic Fat and Brown Adipose Tissue Thermogenesis	194
**Ginger**	NCT02570633	a randomized, double-blind, placebo-controlled clinical trial	not increase energy expenditure in female adults	195
**Creatine**	NCT04086381	a double-blind, randomized, placebo-controlled, cross-over trial	not enhance BAT activation	196
**Mirabegron**	NCT03049462	an open-label study	increases human brown fat, HDL cholesterol, and insulin sensitivity	197
**Salt**	-	a Randomized Placebo-Controlled Study	decreases Diet-Induced Thermogenesis	198
